# Adverse childhood events and risk of diabetes onset in the 1979 National longitudinal survey of youth cohort

**DOI:** 10.1186/s12889-019-7337-5

**Published:** 2019-07-27

**Authors:** E. Anne Lown, Camillia K. Lui, Kate Karriker-Jaffe, Nina Mulia, Edwina Williams, Yu Ye, Libo Li, Thomas K. Greenfield, William C. Kerr

**Affiliations:** 1 Department of Social and Behavioral Sciences, School of Nursing, University of California, 3333 California Street, San Francisco, CA 94118 USA; 2 Alcohol Research Group, Public Health Institute, 6001 Shellmound Ave, Suite 450, Emeryville, CA 94608 USA

**Keywords:** Type 2 diabetes mellitus, Adverse childhood events, Alcohol, Tobacco, Body mass index, Obesity

## Abstract

**Background:**

Type 2 diabetes is a major public health problem with considerable personal and societal costs. Adverse childhood experiences (ACE) are associated with a number of serious and chronic health problems in adulthood, but these experiences have not been adequately studied in relation to diabetes in a US national sample. The association between ACE and poor health can be partially explained by greater risky health behaviors (RHB) such as smoking, heavy alcohol use, or obesity. Few studies have examined ACE in relation to adult onset Type 2 diabetes mellitus (T2DM) taking into account the role of RHB. Using longitudinal data from a representative US population sample followed over 30 years, this study examines the impact of ACE on the risk of diabetes onset.

**Methods:**

Data from the 1982 to 2012 waves of the 1979 National Longitudinal Survey of Youth were analyzed, spanning ages 14 to 56. Bivariate and discrete-time survival models were used to assess the relationships between ACE and RHB including smoking, alcohol use, and obesity, and subsequent onset of diabetes.

**Results:**

T2DM was reported by almost 10% of participants. Over 30% of women and 21% of men reported 2+ ACE events. Women reporting 2–3 or 4+ ACE events were more likely to develop diabetes with the mean number of ACE events being greater in those with diabetes compared to without (1.28 vs.1.05, *p* < .0001). For men there was no significant association between ACE and diabetes onset. For women, ACE was associated with heavy drinking, current smoking, and obesity. For men, ACE was associated with being underweight and daily smoking. In multivariate discrete-time survival models, each additional ACE increased risk of T2DM onset (OR_adj_ = 1.14; 95% CI 1.02–1.26) for women but not for men. The relationship in women was attenuated when controlling for body mass index (BMI).

**Conclusion:**

ACE predicted diabetes onset among women, though this relationship was attenuated when controlling for BMI. Being overweight or obese was significantly more common among women with a history of ACE, which suggests BMI may be on the pathway from ACE to diabetes onset for women.

## Background

Adverse childhood experiences (ACE) are linked to many serious adult health conditions including cancers [1–3], cardiovascular disease or hypertension [2, 4, 5], diabetes [6–9], substance use [2], mental illness [10], disability [11], premature death [12, 13], and significantly greater health care costs [14]. Exposure to ACE can be a long-term determinant of poorer health [15, 16]. ACEs impact later health through biological processes, psychiatric and physical health problems, psychosocial and developmental impacts, and factors related to age and late-life stressors [17].

Type 2 diabetes mellitus (T2DM) is the seventh leading cause of death in the United States (US) [18], affecting almost 29 million Americans [18] and costing the US $245 billion in direct and indirect costs [19]. T2DM is the most prevalent form of diabetes, accounting for 90–95% of diabetes cases in the US [20]. Poorly controlled T2DM can lead to serious health problems including cardiovascular disease (the number one cause of death), lower-limb amputation, blindness, and kidney failure [19]. Careful management of T2DM is critically important in slowing or reversing the course of the disease. T2DM management often focuses on lifestyle interventions such as promoting healthy diet, exercise, losing excess weight, and taking medications.

In the landmark ACE study carried out at Kaiser Permanente in San Diego, CA [2], participants reporting ACE events were more likely to have T2DM. This finding was replicated in subsequent studies [6–9], including a general population, a cross-sectional community survey carried out in ten countries [10] and a large representative sample from ten US states and Washington, DC [7]. A meta-analysis representing 87,000 participants [21] and a systematic review including ten studies (involving population- and community-based cohorts) and over 200,000 people [22] also showed greater risk for T2DM or the development of metabolic abnormalities among respondents reporting ACE. One systematic review described a threshold response in several studies between ACE and T2DM, but the authors noted no clear dose response relationship [23]. This threshold effect also emerged in a cross-sectional study, where 4+ ACE was associated with T2DM, but fewer ACEs were not [23].

Previous research has examined specific ACEs that may raise the risk for T2DM. An Alameda County Study followed a cohort for 34 years and described increased risk for T2DM among those with childhood poverty, especially among women [24]. The Nurse’s Health Study examined 67,853 women and described lifetime physical or sexual abuse as a predictor of T2DM, noting the relationship was partially explained by high BMI among abused women [25].

Previous studies have documented the strong influence of ACE on risky health behaviors (RHBs) in general [26–28], and on obesity [29–35], tobacco use [36–38] and alcohol use [39, 40] specifically. Most studies do not adjust for obesity when examining the relationship between ACE and T2DM [6, 7], with a few exceptions where inclusion of BMI attenuated this relationship [9]. In the aforementioned systematic review [9], the relationship between ACE and T2DM was attenuated in four studies when adjusting for adult poverty and obesity [35, 41–43]. An improved understanding of the complex relationships between adult health behaviors and childhood events has the potential to help patients and providers manage and prevent T2DM.

While some studies describe ACE in relation to the development of T2DM over the life course [24, 25], more research is needed to better characterize a wide range of health behaviors (obesity, tobacco use, and alcohol use) over time. These behaviors may be related to both ACE and poor health outcomes. Previous studies have not controlled for risk factors such as adult poverty, minority race/ethnicity, and educational attainment. The current study aimed to address these gaps by using data from a nationally-representative panel sample starting in adolescence and spanning 38 years. The National Longitudinal Survey of Youth collected information on childhood adverse events, risky health behaviors and diabetes (among other health problems) in a diverse sample of men and women. We expected that ACE would be associated with onset of T2DM, and that the relationship would be partially-explained by health risk behaviors, including obesity, and alcohol use.

## Methods

### Study population

The National Longitudinal Survey of Youth 1979 (NLSY79) is an ongoing study of US individuals born between 1957 and 1964, selected using a stratified, clustered sampling design, that is representative of the non-institutionalized, non-military population of youth ages 14 to 21 in 1979. The initial sample included 3,174 Blacks, 2,002 Hispanics, and 7,510 non-Black/non-Hispanic participants who were interviewed annually between 1979 and 1994 and then every 2 years since 1994. The baseline response rate from NLSY79 was 90%; retention rates were 80% or higher during 16 subsequent assessments but have declined to 71% (77% excluding decedents) in recent assessments [44]. The current sample includes NLSY79 participants who provided data on at least 4 ACE events and on their T2DM status (*N* = 8,377).

### Measures

#### Diabetes

The dependent variable is onset of T2DM. NLSY79 participants completed a detailed health module after they turned 40, and again after they turned 50. Participants were asked, “Have you ever had, or has a doctor ever told you that you have diabetes or high blood sugar?” If they said yes, participants then reported the month and year of onset. Age of diabetes onset was calculated based on respondents’ month and year of birth (provided at the baseline 1979 survey). Included diabetes cases were restricted to onset at age 18 and older.

#### Adverse childhood experiences (ACE)

In the 2012 survey, participants were asked a retrospective set of questions about events/experiences that occurred in childhood (before age 18). We identified the following ACEs: parental death, adverse living situation, living with a mentally ill person, living with a problem drinker, and being physically abused as a child. An adverse living situation between the ages of 0–18 was defined as not living with two biological parents, living with grandparents or foster parents, or living in an orphanage or group home. Living in poverty at the baseline survey in 1979 was also included as an adverse event. The primary ACE measure was the total number of ACE events recorded (range: 0–6). However, in some analyses we used ACE binary indicators of two or more (2+), 3+ and 4+ ACE events compared to 0–1 event, since such threshold measures were consistent with prior studies [36, 45].

#### Risky health behaviors

##### Alcohol use

Respondents were asked about their alcohol use in 1982–1985, 1988, 1989, 1994, 2002, and every other year from 2006 to 2012. We created a time-varying measure of past month alcohol consumption. For years 1982–85, total volume was based on past week consumption of wine, beer and spirits. For 1988–2012, total volume was based on past month usual quantity and frequency for all beverages combined. To account for these measurement differences, weekly volume for 1982–1985 was adjusted downward, and monthly volume for 1988–2012 was adjusted upward, as described elsewhere [46]. We then created categories of total volume: zero drinks, low volume (less than or equal to 14 drinks per week for men or 7 drinks per week for women, according to national low-risk guidelines), risky drinking (more than 14 drinks but less than 28 drinks per week for men or more than 7 drinks and less than 14 drinks per week for women), and high volume (more than 28/14 drinks per week for men/women) [47]. Finally, to address concerns about appropriate non-drinking groups in prior alcohol-related health studies [48, 49], we further divided the no alcohol group into lifetime abstainers and no current alcohol use/former drinkers, as described elsewhere [49]. The low volume drinking group is the reference.

##### Smoking

A set of smoking questions was asked in 1992, 1994, 1998, 2008, 2010, and 2012, including the age when participants started to smoke daily, current daily smoking (yes/no), and cigarettes smoked per day. Summarizing the available data, we created an age of onset of daily smoking and carried it forward from that age, and then grouped smoking status for each year into never daily smoker, former daily smoker, and current daily smoker and referred to in the manuscript as never, former, and current smoker.

##### Obesity

Height was reported in 1981, 1982, 1983, 1985, 2006, 2008, 2010, and 2012. Weight was reported in 1981, 1982, 1985–86, 1988–1990, and every other year from 1992 to 2012. We carried forward height to calculate body mass index (BMI) scores for each available weight year. We calculated BMI as reported by weight in pounds divided by the square of height and then multiplied by a conversion factor of 703 [50]. BMI was coded into underweight (< 18.5), normal (18.5–24.9), overweight (25–29.9), and obese (> = 30), consistent with current recommendations [50].

##### Covariates

We include sex (male and female), foreign- or US-born, and self-reported race/ethnicity: White (reference group), Black, Hispanic, and ‘Other racial/ethnic groups’ (combined, including Asian, Hawaiian, and Pacific Islander, and American Indian). Age was calculated based on interview month and year and birth month and year. Educational attainment by age 25 was coded into less than high school diploma or equivalent, high school graduate, some college, and college degree or more.

We include repeated measures of poverty status (yes/no), marital status (never married, married, separated, divorced, widowed), employment status (unemployed, employed, out of the labor force, active military), and whether the respondent has children (yes/no). These measures were available at each survey year.

### Statistical analyses

The analytic sample included those who had data from the 2012 retrospective ACE questions and had available data on T2DM status in the 40+/50+ health modules. First, we investigated bivariate relationships between the primary predictors (number of ACE events, RHBs) and the outcome (T2DM). We conducted sensitivity analyses to examine both a total ACE score and cut-points of 2+, 3+ or 4+ ACE events versus fewer ACE events (i.e. ACE 3+ vs. 0–2). Testing of multiple cut-points is common in the ACE literature [2, 12, 36, 37, 45, 51–55].

Next, discrete-time survival models were used to model the onset of T2DM. Risk for T2DM starts in 1982, when participants were between the ages of 18 and 25. For those who reported the onset of T2DM before or on 2012 (when ages range from 48 to 55), their risk for T2DM ends in the year the event occurred. For all other participants, the risk period ended in the year when they reached 2012, which was the last survey year available (censoring). To model T2DM risk, time-invariant covariates such as foreign-born and race/ethnicity remained the same across time. Time-varying covariates such as alcohol consumption and employment were first created for the survey years and then were carried forward for the year(s) without interviews, until new data were available from a subsequent interview.

Our discrete-time survival model was implemented by a pooled logistic regression model [56] treating each reconstructed person-year as an observation. To avoid instability of estimation, the linear and quadratic terms for time (defined as the number of years from 1982) were added into our discrete-time survival model with the other time-variant and invariant covariates. No formal mediation testing was conducted given the categorical nature of the RHBs. Rather we implemented a series of models first adjusting for ACE and covariates (Model 1), then examining changes in ACE coefficient after adding in BMI (Model 2), and again after adding in alcohol and smoking (Model 3). Separate models were estimated for men and women and for White, Black, and Hispanic respondents (also stratified by sex). For the NLSY, survey weights are available for each year to adjust the sample to its original sampling frame, to be representative of US youth demographics in 1979, and account for attrition. All analyses were conducted in Stata version 14.2.

## Results

### Sample characteristics

Table 1 shows sample characteristics stratified by sex. Females constitute roughly 52% (*N* = 4,328) of the sample. Prevalence of diabetes was approximately 10% for both women and men in this sample. Diabetes onset ranged from age 18 to age 52. Only two cases of diabetes were diagnosed in participants who were aged 18–20, (0.2% of all diabetes cases). Given that 90% of all diabetes cases are T2DM, and the fact that the vast majority of Type 1 diabetes is diagnosed in young people age 1–15, the authors have made the assumption that most all diabetes cases in this study are T2DM [57]. The most commonly reported ACE for both men and women was growing up living in a non-traditional situation with 35.7 and 31.7% respectively. In 1982, the mean participant age was 21 years old. The sample was predominantly White (61% for females and 64% for males). Between 12 and 14% reported less than a high school education and 21% reported college or more. Almost one-third of females (30%) reported two or more ACEs compared to 21% of males. Poverty was experienced by 14% of female and 10% of male participants at some point in the course of the study.

**Table 1:** Sample characteristics of NLSY79 cohort

Weighted % (N)	Female (*n* = 4,328)		Male (*n* = 4,049)	
Diabetes	9.70%	(431)	10.2%	(367)
Age of Diabetes Onset (Mean Years)	43.5		45.3	
Time-Invariant Variables				
Adverse Childhood Events				
Childhood Poverty	14.3%	(960)	12.6%	(767)
Parental Death	8.1%	(353)	7.9%	(298)
Non-Traditional Living Situation	35.7%	(1,457)	31.7%	(1,172)
Lived w/Mentally Ill Person	11.3%	(358)	5.0%	(141)
Lived w/Problem Drinker	22.8%	(794)	15.7%	(506)
Physical Abuse	16.8%	(621)	12.7%	(417)
2+ Ace Events				
No	69.6%	(2,384)	76.9%	(2,378)
Yes	30.4%	(1,342)	21.1%	(954)
Age in 1982 (Mean)	20.72		20.53	
Race/Ethnicity				
White	60.9%	(1,477)	63.5%	(1,382)
Black	15.2%	(1,125)	14.3%	(983)
Hispanic	6.4%	(664)	5.9%	(580)
Other	17.5%	(445)	16.4%	(372)
U.S. Born				
Yes	95.9%	(3,479)	96.2%	(3,114)
Education Attainment				
Less than High School	11.8%	(590)	13.6%	(621)
High School	44.9%	(1,652)	44.7%	(1,509)
Some College	22.9%	(889)	20.5%	(661)
College or more	20.5%	(593)	21.3%	(536)
Time-Varying Variables (1982–2012)				
Children (Mean)	1.60		0.90	
Poverty Status				
No	86.1%		89.7%	
Yes	14.0%		10.3%	
Marital Status				
Never Married	22.3%		31.5%	
Married	58.4%		54.9%	
Separated	4.3%		2.9%	
Divorced	14.1%		10.5%	
Widowed	1.0%		0.2%	
Employment Status				
Employed	75.3%		85.9%	
Unemployed	2.0%		2.9%	
Out of Labor Force	22.4%		8.8%	
Active Services	0.3%		2.5%	

### Bivariate relationships

In evaluating the specific ACE items, (Table 2) childhood poverty and parental death were significantly associated with T2DM (OR_crude_ 1.54; 95% CI 1.18, 2.01) and (OR_crude_ 2.18; 95% CI, 1.50, 3.18) respectively. Women reporting 2 ACEs and 4+ ACEs were significantly more likely to have T2DM compared to those who reported fewer ACEs. Women with T2DM had significantly higher mean ACE scores compared to women without T2DM, (1.28 vs. 1.05, *p* < .0001). Among men, T2DM was associated with having 4+ ACEs, (OR_crude_ = 2.51; 95% CI 1.12, 5.63) but not with any individual ACE items.

**Table 2:** Odds ratio for adverse childhood events by diabetes status and mean number of ACE for men and women with diabetes

	Female (*n* = 3,726)				Male (*n* = 3,332)			
	%	n	OR	95% CI	%	n	OR	95% CI
Childhood Poverty								
No	9.2%	(279)			10.1%	(253)		
Yes	13.6%	(136)	1.54*	(1.18, 2.01)	10.6%	(89)	1.05	(0.76, 1.47)
Parental Death								
No	8.9%	(373)			10.3%	(331)		
Yes	17.6%	(56)	2.18*	(1.50, 3.18)	9.0%	(35)	0.86	(0.53, 1.40)
Non-Traditional Living Situation								
No	9.3%	(238)			9.9%	(222)		
Yes	10.4%	(180)	1.13	(0.87, 1.46)	11.0%	(131)	1.12	(0.84, 1.49)
Lived w/Mentally Ill Person								
No	9.4%	(381)			10.0%	(345)		
Yes	11.5%	(49)	1.24	(0.84, 1.83)	15.1%	(21)	1.6	(0.92, 2.80)
Lived w/Problem Drinker								
No	9.4%	(331)			10.0%	(310)		
Yes	10.4%	(99)	1.11	(0.83, 1.50)	11.3%	(57)	1.15	(0.80, 1.65)
Physical Abuse								
No	9.3%	(339)			9.8%	(311)		
Yes	11.5%	(89)	1.27	(0.93, 1.73)	13.2%	(56)	1.41	(0.96, 2.07)
Sum of Adverse Childhood Events								
0 ACE	7.9%	(116)			9.8%	(148)		
1 ACE	9.9%	(135)	1.28	(0.93, 1.76)	9.7%	(104)	0.99	(0.72, 1.38)
2 ACE	11.4%	(104)	1.50*	(1.06, 2.12)	10.8%	(73)	1.12	(0.77, 1.64)
3 ACE	10.3%	(43)	1.34	(0.85, 2.12)	11.3%	(29)	1.18	(0.67, 2.06)
4+ ACE	16.1%	(33)	2.23*	(1.32, 3.75)	21.3%	(13)	2.51*	(1.12, 5.63)
Mean Adverse Childhood Events by Diabetes Prevalence								
	Diabetes-Female				Diabetes-Male			
	Yes	No	*P*-value		Yes	No	P-value	
Mean ACE Score	1.28	1.05	<.0001		0.93	0.82	0.062	

Notes: p-value * *p* < 0.05, **p < 0.01, ***p < .001

Overweight and obese women had higher ACE scores compared to normal weight women. For men, the mean number of ACEs was significantly greater for those who were underweight compared to overweight or obese BMI categories (Table 3). For women, the mean number of ACEs was significantly greater for high volume, former drinkers, and lifetime abstainers compared to low volume drinkers. Mean ACE events also were greater for women who were current smokers compared to never or former smokers. Men who were current smokers had greater mean ACEs compared to former and never smokers.

**Table 3:** Mean adverse childhood events by health behavior

	Female			Male		
	Mean	95% CI	*p*-value	Mean	95% CI	*p*-value
Body Mass Index						
Underweight	1.05	(1.02, 1.12)	< 0.001	1.01	(0.91, 1.11)	< 0.001
Normal Weight	0.99	(0.98, 1.01)		0.84	(0.83, 0.86)	
Overweight	1.06	(1.05, 1.09)		0.80	(0.79, 0.81)	
Obese	1.20	(1.18, 1.22)		0.85	(0.84, 0.87)	
Alcohol Use						
Lifetime Abstainer	1.07	(1.06, 1.11)	< 0.001	0.75	(0.72, 0.78)	< 0.001
Former Drinker	1.15	(1.14, 1.17)		0.97	(0.96, 0.99)	
Low ≤14/7 per wk	0.98	(0.97, 0.99)		0.76	(0.75, 0.77)	
Risky (> 14/7 per week)	1.02	(0.99, 1.05)		0.84	(0.82, 0.87)	
High Volume (> 28/14 per week)	1.22	(1.18, 1.28)		0.97	(0.94, 1.01)	
Smoking Status						
Never Daily Smoker	0.86	(0.85, 0.88)	< 0.001	0.70	(0.69, 0.71)	< 0.001
Former Daily Smoker	1.17	(1.16, 1.20)		0.91	(0.89, 0.93)	
Current Daily Smoker	1.26	(1.25, 1.28)		0.98	(0.97, 0.99)	

**Fig. 1:**
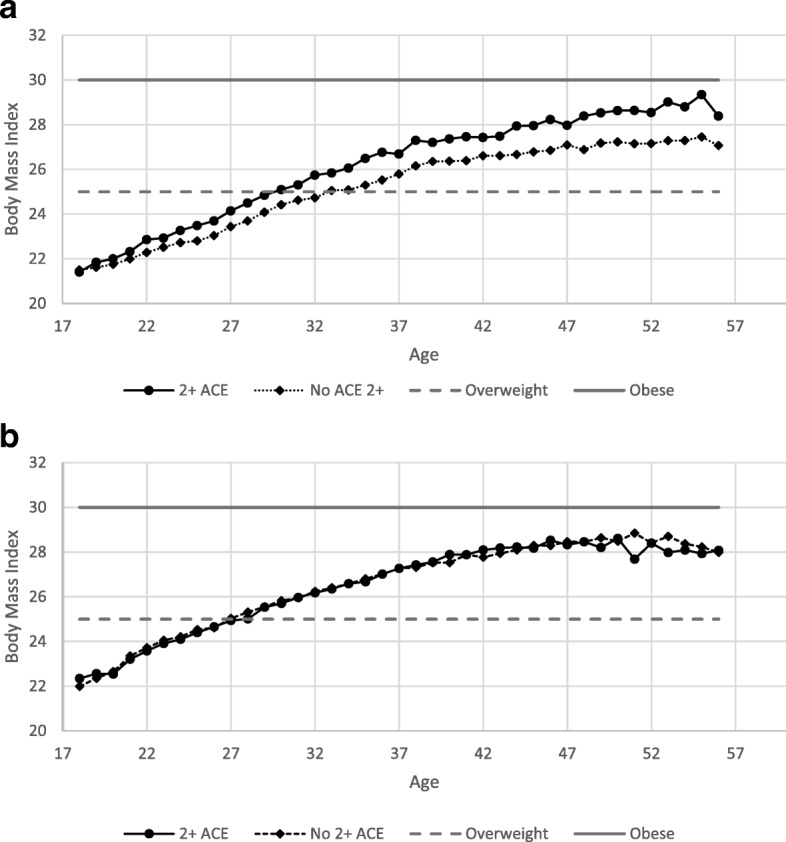
**a** Female Body Mass Index by Age and Adverse Childhood Events. **b** Male Body Mass Index by Age and Adverse Childhood Events

Figure 1 shows increasing BMI with age (as expected), with the total BMI being significantly higher among women who reported 2 + ACE (**a**) and overweight status being reached at a younger age among those with 2 + ACE (mean age 29) compared to those with < 2 ACE. Those with < 2 ACE became overweight later (mean age 33). Among men, BMI was not different by 2 + ACE status (**b**).

### Survival models

T2DM onset was predicted using discrete-time survival models controlling for time-invariant and time-varying covariates in Model 1 (Total Effect Model), with the addition of BMI adjustments in Models 2 (Direct Effects BMI_adj_) and all 3 RHBs in Model 3 (Direct Effects-RHB_adj_ (Table 4).

**Table 4:** Discrete-time models of diabetes onset, NLSY79

	Female						Male					
	Model 1 Total effect		Model 2 Direct effect BMI_adj_		Model 3 Direct effect- RHB_adj_		Model 1 Total effect		Model 2 Direct effect BMI_adj_		Model 3 Direct effect- RHB_adj_	
	OR	95% CI	OR	95% CI	OR	95% CI	OR	95% CI	OR	95% CI	OR	95% CI
Time	1.29*	(1.19, 1.39)	1.23*	(1.13, 1.33)	1.22*	(1.13, 1.33)	1.51*	(1.36, 1.68)	1.44*	(1.29, 1.61)	1.46*	(1.31, 1.63)
Time-squared	0.99*	(0.99, 1.00)	1.00*	(0.99, 1.00)	1.00*	(0.99, 1.00)	0.99*	(0.99, 0.99)	0.99*	(0.99, 1.00)	0.99*	(0.99, 0.99)
ACE Score (0–6)	1.14*	(1.02, 1.26)	1.10	(1.00, 1.22)	1.11^t^	(1.00, 1.23)	1.09	(0.95, 1.25)	1.09	(0.94, 1.25)	1.10	(0.96, 1.27)
Age	1.10*	(1.05, 1.15)	1.10*	(1.05, 1.15)	1.10*	(1.05, 1.16)	1.19*	(1.13, 1.25)	1.19*	(1.13, 1.26)	1.21*	(1.14, 1.28)
Born in US	1.09	(0.60, 1.99)	1.01	(0.56, 1.84)	1.04	(0.58, 1.88)	0.63	(0.34, 1.15)	0.59	(0.32, 1.10)	0.56	(0.30, 1.05)
Poverty status	1.41*	(1.04, 1.90)	1.42*	(1.05, 1.91)	1.39*	(1.03, 1.87)	1.02	(0.65, 1.59)	1.10	(0.69, 1.75)	1.08	(0.68, 1.73)
Has children	0.91	(0.83, 1.00)	0.91*	(0.83, 1.00)	0.91	(0.83, 1.00)	0.96	(0.85, 1.09)	0.95	(0.83, 1.07)	0.95	(0.83, 1.08)
Race (Ref. White)												
Black	1.46*	(1.10, 1.94)	1.06	(0.81, 1.40)	1.02	(0.77, 1.35)	0.95	(0.70, 1.30)	0.90	(0.66, 1.23)	0.90	(0.66, 1.22)
Hispanic	1.86*	(1.33, 2.59)	1.53*	(1.12, 2.11)	1.54*	(1.12, 2.13)	1.05	(0.70, 1.58)	0.93	(0.61, 1.42)	0.93	(0.60, 1.44)
Other	1.32	(0.92, 1.88)	1.28	(0.89, 1.82)	1.27	(0.88, 1.82)	1.01	(0.69, 1.49)	1.03	(0.69, 1.52)	0.96	(0.64, 1.45)
Marital status (Ref. Married)												
Never Married	0.93	(0.64, 1.34)	0.81	(0.57, 1.16)	0.81	(0.57, 1.15)	1.35	(0.91, 2.01)	1.49	(1.00, 2.21)	1.54*	(1.02, 2.30)
Separated	0.85	(0.56, 1.29)	0.79	(0.52, 1.20)	0.84	(0.55, 1.27)	0.80	(0.35, 1.82)	0.93	(0.42, 2.10)	1.00	(0.44, 2.24)
Divorced	0.86	(0.62, 1.19)	0.96	(0.69, 1.32)	0.98	(0.71, 1.37)	1.32	(0.88, 1.97)	1.42	(0.94, 2.14)	1.50	(0.99, 2.27)
Widowed	0.44*	(0.20, 0.98)	0.40*	(0.18, 0.90)	0.41*	(0.18, 0.93)	4.68*	(1.54, 14.18)	4.09*	(1.53, 10.95)	4.05*	(1.61, 10.22)
Employment (Ref. Employed)												
Unemployed	1.05	(0.51, 2.15)	1.04	(0.51, 2.13)	1.03	(0.50, 2.09)	1.32	(0.68, 2.54)	1.27	(0.66, 2.46)	1.29	(0.66, 2.52)
Out of Labor Force	1.44*	(1.07, 1.94)	1.46*	(1.08, 1.97)	1.38*	(1.02, 1.86)	0.93	(0.56, 1.53)	0.89	(0.53, 1.50)	0.82	(0.48, 1.39)
Active Military	2.17	(0.29, 15.98)	3.30	(0.52, 20.93)	3.32	(0.50, 22.05)	1.42	(0.48, 4.25)	1.83	(0.61, 5.49)	0.45	(0.10, 1.99)
Education (Ref. <HS Grad)												
HS Grad	1.14	(0.79, 1.63)	1.21	(0.85, 1.73)	1.21	(0.85, 1.72)	1.04	(0.73, 1.47)	1.01	(0.72, 1.43)	1.06	(0.74, 1.53)
Some College	1.49*	(1.01, 2.19)	1.81*	(1.24, 2.65)	1.89*	(1.28, 2.80)	0.94	(0.62, 1.43)	0.91	(0.60, 1.38)	0.99	(0.64, 1.53)
College or more	0.93	(0.57, 1.51)	1.30	(0.80, 2.10)	1.35	(0.82, 2.24)	0.51*	(0.31, 0.85)	0.62	(0.38, 1.03)	0.72	(0.43, 1.22)
BMI (Ref. Normal Weight)												
Underweight			3.87*	(1.73, 8.67)	4.08*	(1.80, 9.21)			0.75	(0.10, 5.73)	0.79	(0.10, 6.09)
Overweight			2.89*	(1.93, 4.35)	3.00*	(1.99, 4.52)			2.38*	(1.45, 3.93)	2.52*	(1.51, 4.21)
Obese			8.97*	(6.32, 12.74)	8.90*	(6.19, 12.79)			7.57*	(4.67, 12.28)	7.84*	(4.79, 12.84)
Alcohol Use (Ref. Low use [<=14/7 drinks per wk])												
Lifetime Abstainer					1.56*	(1.07, 2.27)					1.35	(0.74, 2.48)
Former Drinker					1.26	(0.95, 1.67)					1.20	(0.89, 1.61)
Risky Drinker (> 14/7 per week)					1.11	(0.59, 2.06)					0.76	(0.43, 1.34)
High Volume Drinker (> 28/14 per week)					1.68	(0.80, 3.51)					1.11	(0.55, 2.27)
Smoking Status (Ref. Never Daily Smoker)												
Former Daily Smoker					0.87	(0.62, 1.22)					0.96	(0.68, 1.35)
Current Daily Smoker					0.94	(0.69, 1.29)					1.16	(0.84, 1.60)

*significance at the *p* < 0.05 level, ^t^ = 0.05

Among women, in the Total Effect, Model 1, mean ACE score significantly predicted T2DM onset (OR_adj_ = 1.14; 95% CI 1.02, 1.26)). Adjustment for BMI and all three RHBs added in Models 2 and 3 reduced the effect size between ACE and T2DM. In model 2-Direct Effects-BMI_adj_, BMI was added, resulting in a slight decrease in the effect size (OR_adj_ = 1.10; 95% CI 1.00, 1.22) between ACE and T2DM and a reduction from significance to marginal significance (*p* = 0.06). BMI was a strong predictor for T2DM in underweight (OR_adj_ = 3.87; 95% CI 1.73, 8.67), overweight (OR_adj_ = 2.89; 95% CI 1.93, 4.35), and obese (OR_adj_ = 8.97; 95% CI 6.32, 12.74) groups compared to the normal group. In Model 3 Direct Effects-RHB_adj_, female lifetime abstainers showed increased risk for T2DM (OR_adj_ = 1.56; 95% CI 1.07, 2.27) compared to their low-risk drinking counterparts. ACE effect size was reduced (OR_adj_ = 1.11; 95% CI 1.00, 1.23) and remained significant, *p* = 0.05). Smoking did not predict T2DM onset. Other significant risk factors for T2DM onset among women include older age, living in poverty, being out of the labor force, attending only some college, and being Hispanic or Black compared to being White. Being widowed was protective.

Among men, ACE did not predict T2DM onset in any models. In Model 2- Direct Effects-BMI_adj_, being overweight (OR_adj_ = 2.38; 95% CI 1.45, 3.93) or obese (OR_adj_ = 7.57; 95% CI 4.67, 12.28), was a strong predictor of T2DM but the introduction of BMI did not alter the effect size between ACE and T2DM. Alcohol and smoking were not predictive of T2DM in Model 3-Direct Effects-RHB_adj_.

Given the significant racial/ethnic differences in T2DM risk among women seen in Table 4 Model 1-Total Effect, we conducted exploratory analyses among women only, stratifying by race (Table 5). ACE was not a significant predictor of T2DM onset for White, Black, Hispanic or “Other” women. In all cases, being overweight or obese was a significant predictor for T2DM and tobacco and alcohol use were not significant predictors except in the case of “Other” women where former drinkers were more likely to have T2DM (OR_adj_ = 2.35, *p* = 034).

**Table 5:** Race and sex-stratified models of diabetes onset, NLSY79 – model showing direct effect- RHB_adj_

	Female							
	White		Black		Hisp		Other	
	OR	*p*-value	OR	*p*-value	OR	*p*-value	OR	*p*-value
Time	1.22*	0.002	1.31*	0.000	1.34*	0.001	1.14	0.131
Time-squared	1.00*	0.012	0.99*	0.000	0.99*	0.001	1.00	0.176
ACE-Score (0-6)	1.10	0.221	1.04	0.668	1.05	0.569	1.23	0.186
Age	1.10*	0.014	1.12*	0.002	1.09*	0.046	1.10	0.209
Born in US	0.95	0.927			1.46	0.159	0.44	0.258
In Poverty	1.00	0.992	1.83*	0.006	1.80*	0.032	2.10*	0.041
Has children	0.90	0.238	0.87*	0.025	0.87	0.168	1.03	0.783
Marital Status (Ref. Married)								
Never Married	0.76	0.398	0.80	0.323	0.77	0.452	1.17	0.775
Separated	0.13*	0.006	0.97	0.911	1.22	0.592	1.57	0.471
Divorced	1.21	0.445	1.00	0.998	0.73	0.303	0.65	0.366
Widowed	--	--	1.06	0.918	0.59	0.473	0.32	0.231
Employment (Ref. Employed)								
Unemployed	1.32	0.653	1.12	0.771	0.46	0.439	--	--
Out of Labor Force	1.62*	0.031	0.60*	0.029	1.38	0.172	1.73	0.167
In Active Forces	8.61*	0.023	--	--	--	--	--	--
Education (Ref. <HS Grad)								
HS Grad	1.10	0.774	1.08	0.756	1.23	0.453	1.88	0.218
Some College	2.32*	0.018	1.08	0.784	2.07*	0.032	2.10	0.239
College or more	1.29	0.542	0.81	0.578	1.27	0.621	2.81	0.148
Alcohol Use (Ref low <=14/7 per wk)								
Lifetime Abstainer	1.77	0.054	0.96	0.885	1.44	0.294	3.09	0.086
Former Drinker	1.19	0.449	1.20	0.352	0.67	0.187	2.35*	0.034
Risky Drinker (>14/7 per week)	0.97	0.949	1.08	0.862	0.89	0.819	2.18	0.376
High Volume Drinker (>28/14 per week)	2.43	0.055	0.87	0.824	2.26	0.144	--	--
Smoking Status (Ref. Never Daily Smoker)								
Former Daily Smoker	0.78	0.380	0.93	0.784	1.10	0.709	1.08	0.883
Current Daily Smoker	0.95	0.841	0.87	0.535	1.15	0.649	0.96	0.934
BMI (Ref. Normal Weight)								
Underweight	4.48*	0.005	1.72	0.603	--	--	4.88	0.054
Overweight	2.96*	0.000	2.99*	0.003	2.11*	0.036	3.57*	0.010
Obese	10.25*	0.000	8.31*	0.000	7.95*	0.000	6.58*	0.000
Intercept	0.00	0.000	0.00	0.000	0.00	0.000	0.00	0.000

*significance at the *p*<0.05 level

## Discussion

The current study used nationally representative longitudinal data across 30 years to examine ACE in relation to T2DM onset after age 17, together with RHB. Previous literature has hypothesized that RHB, which are more common in people reporting ACE (i.e. high BMI, smoking, or heavy alcohol use), might explain increased risk for numerous poor health outcomes in those with an ACE. Many ACE-T2DM studies have not included RHB with some notable exceptions [25, 35, 42, 43], and to date, findings have focused on BMI as a risk factor. In the current study, with inclusion of a larger number of adult health behaviors, survival models provided confirmatory evidence that ACE predicts T2DM onset among women, even while controlling for other RHBs (*p* = .05). This finding is consistent with previous studies [24], noting that physical or sexual abuse predicted T2DM and that the relationship, as in the present study, was partially explained by high BMI in the abused women [25]. While ACEs in the present study were associated with greater RHB, results suggest that BMI may remain the most important mechanism for the association between ACE and T2DM in women, one that also appears to contribute to racial/ethnic disparities in women’s T2DM risk.

T2DM was associated with individual adverse events among women including childhood poverty, and parental death, as well as with 2+ ACE events. Among men, 4+ ACE predicted T2DM in bivariate models only. In multivariate models, mean ACE score predicted T2DM onset in women, but not in men. Further analyses showed that RHBs, and BMI in particular, were higher among participants reporting 2+ ACE. When we accounted for these RHBs, ACE was more weakly associated with T2DM in women. Despite inclusion of additional behavioral risks, BMI emerged as the strongest predictor of T2DM onset, with women’s lifetime abstention associated with a marginally increased risk.

Numerous studies including a Danish national cohort [58] and several meta-analyses [59–61] have described the protective effect of light to moderate drinking on T2DM, with some exceptions among women [61, 62]. Similar to these studies, the present study identified higher risk for T2DM among women lifetime abstainers compared to light drinkers, showing a protective effect for light drinking among women only. While studies have documented a beneficial effect of light alcohol use on T2DM onset, a large case-cohort study concluded that the increased risk was mostly explained by confounding factors, cautioning that people should not take up drinking with the hope that it could prevent T2DM [63]. A meta-analysis representing 1.9 million participants concluded that a reduction in risk among drinkers (increased risk in non-drinkers) was only apparent for women [61]. This finding was consistent with the present study where women lifetime abstainers had higher risk for T2DM (low drinking had a protective effect) even controlling for ACE and other confounders.

Smoking status showed a graded pattern of association with ACE where never smokers had the fewest ACE events and current smokers had the most events. This finding is consistent with other ACE studies [37]. While smoking has been shown to increase the risk for T2DM in previous studies [64, 65], in the present sample this relationship was not evident. Among people with T2DM smoking complicates medical management and increases health risks related to cardiovascular disease and peripheral neuropathies [65, 66].

The strong relationship between ACE and obesity found in women overall was present in each racial/ethnic group. This finding suggests that women of all backgrounds may be more likely to gain weight possibly as an internalizing attempt to cope with psychological stress or trauma following ACE [9, 67] where eating is an attempt to regulate emotions in the face of stress [68]. Weight gain may further contribute to T2DM risk [69]. Serious stress in childhood is known to compromise the hypothalamic-pituitary-adrenal axis and activate the sympathetic nervous system leading to difficulty with emotion regulation, obesity, and increase risk for metabolic syndrome [70].

Racial/ethnic disparities in women’s T2DM risk were also documented in this sample, as in prior studies. Black women, known to be at greater risk for T2DM in general [24], showed higher risk for T2DM relative to white women when adjusting for ACE and demographic risk factors, but this difference disappeared after accounting for BMI. By contrast, Hispanic women’s elevated risk for T2DM remained significant [71], albeit reduced, after accounting for BMI. While BMI cannot easily be separated out from access to healthy foods, outdoor physical activities, and socioeconomic status, the current study provides cautionary evidence that reducing BMI among Black and Hispanic women with a history of ACE may be important in preventing racial/ethnic disparities in T2DM, along with addressing additional factors thought to contribute to these disparities [72]. The present findings are consistent with previous studies where early childhood poverty, a factor closely related to poorer nutritional status, was a predictor for adult T2DM [6, 22]. Our results from race-stratified models suggest that for racial/ethnic minority women, the risk associated with childhood poverty could be further compounded by adult poverty, as the latter was a significant contributor to T2DM in racial/ethnic minority women. Previous research documenting an association between health behaviors and higher rates of T2DM can be explained, in part, by environmental factors such as poverty, crime, and segregated housing that are associated with poor access to healthy food and limited opportunities for physical activity [72–74].

The current study has a number of strengths including the large sample size, use of longitudinal data covering over 35 years starting while participants were adolescents, and inclusion of health behavior measures over time. In addition, the NLSY included the onset of several serious health outcomes including diabetes. This data provided a unique opportunity to examine the emergence of risk behaviors in the years following ACE and to track the onset of diabetes in relation to these health behaviors over time.

As with any study, there are some limitations. While a strength was use of time-varying measures of health behaviors such as drinking volume, another measures of alcohol use, binge drinking, that is commonly used, is not assessed here, thus findings are specific to the alcohol covariates used [75]. The exclusion of certain ACE questions, i.e., child emotional and sexual abuse, may mean that the available ACE variable was less severe than ACE variables used in other studies. Previous studies have found that childhood sexual abuse is a strong predictor for adverse health outcomes [76–78]. However, the present study did include childhood physical abuse. Child physical abuse was the only ACE event that was consistently related to all six serious chronic diseases studied, as reported in a ten country study, thus capturing a great deal of the variance in explaining subsequent harms from ACE [10]. Recent psychometric evaluations documented that use of a 2-item measure that includes parental alcohol and *childhood emotional abuse items* can correctly identify people with ACE experiences with 90% sensitivity [79]. The omission of sexual and emotional abuse may result in an underestimate of the actual impact of adverse events on long-term health and diabetes. Some previous studies examined levels of ACE and noted a threshold effect after 4+ ACE [23]. This finding was confirmed for both women and men in the present study. The present study uses ACE as a continuous variable with levels ranging from 0 to 6, allowing assessment of ACE as a gradient and avoiding results that can fluctuate between categories. Finally, NLSY questions about diabetes onset did not differentiate between Type 1 and Type 2 diabetes. However, Type 1 diabetes onset is rare above age 20. The current data included only 0.2% of diabetes cases that were age 20 or younger and 2.9% of cases were age 25 or below. Thus, results from the study are unlikely to be different in the unlikely event that some of these cases were type 1 diabetes. Finally, analyses that reported differences in effect sizes as an assessment of mediation rely on many assumptions as highlighted by Vanderweele [80, 81], (e.g., no interaction between exposure and mediator, no unmeasured confounding, no mediator-outcome confounding). Those assumptions are common to any kind of regression-based mediation analyses. Violation of those assumptions may affect the interpretation of our results.

## Conclusion

The present study describes a graded relationship between ACE and T2DM among women, with additional findings showing that the relationship may be strongly influenced by the higher risk for overweight and obese status among women with a history of ACE. To prevent T2DM among women at risk, it is recommended that women receive counseling to reduce their diabetes risk consistent with national recommendations [82–86] addressing physical activity, diet, and weight loss and recommendations that programs be tailored to address the needs of Black [87] and Hispanic women [88]. Additional screening for ACE should occur so that women with a history of ACE receive combined psychological counseling along with traditional diabetes prevention.

## Data Availability

The data analyzed during the current study were drawn from the National Longitudinal Surveys public-use data for the 1979 cohort available at no cost via the Investigator website, (https://www.nlsinfo.org/investigator/pages/login.jsp?p=timeout) an online search and extraction site that enables the user to review NLSY variables and create individual data sets. Information on the data is described here: https://www.bls.gov/nls/nlsy79.htm.

## Abbreviations

ACE: Adverse childhood experiences
BMI: Body mass index
NLSY79: The National Longitudinal Survey of Youth 1979
OR: Odds ratio
T2DM: Type 2 diabetes mellitus
